# Grasping Embodiment: Haptic Feedback for Artificial Limbs

**DOI:** 10.3389/fnbot.2021.662397

**Published:** 2021-05-26

**Authors:** Charles H. Moore, Sierra F. Corbin, Riley Mayr, Kevin Shockley, Paula L. Silva, Tamara Lorenz

**Affiliations:** ^1^Department of Psychology, Center for Cognition, Action, & Perception, University of Cincinnati, Cincinnati, OH, United States; ^2^Department of Electrical Engineering and Computer Science, University of Cincinnati, Cincinnati, OH, United States; ^3^Department of Mechanical and Materials Engineering, University of Cincinnati, Cincinnati, OH, United States

**Keywords:** sense of embodiment, upper-limb prostheses, prosthesis abandonment, vibrotactile feedback, rubber hand illusion paradigm

## Abstract

Upper-limb prostheses are subject to high rates of abandonment. Prosthesis abandonment is related to a reduced sense of embodiment, the sense of self-location, agency, and ownership that humans feel in relation to their bodies and body parts. If a prosthesis does not evoke a sense of embodiment, users are less likely to view them as useful and integrated with their bodies. Currently, visual feedback is the only option for most prosthesis users to account for their augmented activities. However, for activities of daily living, such as grasping actions, haptic feedback is critically important and may improve sense of embodiment. Therefore, we are investigating how converting natural haptic feedback from the prosthetic fingertips into vibrotactile feedback administered to another location on the body may allow participants to experience haptic feedback and if and how this experience affects embodiment. While we found no differences between our experimental manipulations of feedback type, we found evidence that embodiment was not negatively impacted when switching from natural feedback to proximal vibrotactile feedback. Proximal vibrotactile feedback should be further studied and considered when designing prostheses.

## Introduction

Despite decades of public and private research into the development of upper-limb prosthetics, a significant portion of individuals who are prescribed upper-limb prostheses become unwilling and subsequently opposed to wearing them—a problem known as prosthesis abandonment (Biddiss and Chau, 2007a). Even the most expensive category of prostheses, electric prostheses, was estimated in a large longitudinal study to have a rejection rate of 23% (Biddiss and Chau, 2007b), and by a more recent study to have a rejection rate off 18% (Resnik et al., 2020). One of the core issues resulting in prosthesis abandonment is a reduced sense of embodiment, i.e., the sense of self-location, agency, and ownership that humans feel in relation to their bodies and body parts (Murray, 2008). If a prosthesis does not evoke a sense of embodiment, the user is less likely to view it as useful and integrated with their body. Besides the risk of prosthesis abandonment, sense of embodiment is crucial for individuals with acquired limb loss and congenital limb deficiency, as lack of sense of prosthesis embodiment is also connected to higher levels of depression, activity reduction, and lower levels of social integration (Murray, 2004).

Reintroducing closed loop feedback modalities such as haptic feedback is a commonly cited method to improve the sense of embodiment and overall usability of prostheses (Marasco et al., 2011; Saunders and Vijayakumar, 2011; Page et al., 2018). State-of-the-art neural prostheses using implanted peripheral nerve interfaces have made vast improvements in motor control and have begun to offer forms of haptic feedback through direct nerve stimulation (Cuberovic et al., 2019; Middleton and Ortiz-Catalan, 2020), but safety concerns have limited this feedback's strength and efficacy (Günter et al., 2019). Applying vibrotactile feedback to a residual area on a lost or congenially deficient limb that is coupled with pressure sensitive elements at key locations on a prosthesis may be a safe, cheap, and effective alternative to direct peripheral nerve stimulation in the restoration of haptic feedback in prosthetic devices. Our study contributes to the investigation of this idea by replicating a rubber hand illusion effect in immersive virtual reality to explore how applying proximal vibrotactile feedback affects the sense of embodiment of a virtual arm during grasping activities.

### Sense of Embodiment and the Virtual Hand Illusion

Sense of embodiment refers to the sense of self-location, agency, and ownership that humans feel in relation to their bodies and body parts (Carruthers, 2008; Kilteni et al., 2012; Gouzien et al., 2017; Frohner et al., 2019). Interestingly, sense of embodiment has been shown to be elastic, and can be manipulated in an individual by altering the sensory information they have access to. Sense of embodiment can be rapidly and reliably induced with an artificial hand via the rubber hand illusion paradigm. The rubber hand illusion was first empirically investigated by Botvinick and Cohen (1998). Participants sat at a table that visually obscured their left hand but showed an artificial rubber hand in lieu of the obscured hand directly in front of the participant. To induce the original illusion, the rubber hand and the obscured hand are simultaneously brushed to couple visual and haptic feedback. This synchronous multimodal stimulation results in a reportedly strong sense of ownership of the rubber hand and a proprioceptive drift—a perceived change in location of one's real, but obscured, hand toward the location of the artificial hand—when asked to blindly point to the tip of one's own obscured finger with one's visible hand. Thus, for the rubber hand illusion to be successful, visual feedback of the rubber hand must be coupled with haptic feedback as perceived on the obscured real hand. This causes proprioceptive drift toward the location of what is seen: the artificial rubber hand. When the rubber hand illusion is in effect, the participant not only reports that the artificial hand feels like it is a part of them, but that it seems to replace their existing hand, indicating that their sense of embodiment has shifted to the artificial hand.

Interestingly, induction of the rubber hand illusion paradigm is not limited to a coupling of visual and haptic feedback. It can also be achieved by coupling visual feedback with proprioceptive information that results from movement of the obscured hand (Dummer et al., 2009; Kammers et al., 2009). Importantly, the strength of the illusion and its effect on sense of embodiment depends on the temporal synchrony of the visual feedback with another modality, such as haptic or proprioceptive feedback. If, for example, the participant taps their real fingers, the artificial hand must exhibit congruent movements simultaneously for the illusion to be induced (Arata et al., 2014).

Since the original experiment, the rubber hand illusion has been reproduced and modified in various scenarios, including replacing the real hand with artificial hands in virtual and augmented reality, or robotic hands (Suzuki et al., 2013; Aymerich-Franch et al., 2017; Huynh et al., 2019). However, all studies have consistently shown that besides visual information, at least one mode of synchronous sensory information must couple the artificial hand to the unseen hand.

### Haptic Feedback

The human hand has one of the highest densities of mechanoreceptors in the body, and the sense of touch, or haptic feedback, is useful in many everyday tasks. Lack of haptic feedback is associated with a myriad of general problems, including inability to sense limb movement and position, major impairment in skilled performance, and abnormal and spontaneous movements (Johansson and Westling, 1987; Augurelle, 2002; Hager-Ross and Johansson, 2004). However, the majority of affordable and readily available prostheses, such as myoelectric and body powered prostheses, offer no replacement for haptic feedback when the prosthetic is physically contacted, requiring amputees to rely entirely on visual feedback. Neural prostheses are beginning to offer forms of haptic feedback through stimulation of reinnervated nerves. For the time being, however, signal strength and sustainability are both limited by safety concerns (Günter et al., 2019), resulting in users reporting an inability to sense degree of grasping pressure and no meaningful sense of “losing the grip of something” (Middleton and Ortiz-Catalan, 2020). Despite this limited degree of haptic feedback reintroduction, neural prosthesis users have reported an increase in their sense of embodiment of their prosthetic after switching from a non-neural to a neural prosthesis (Cuberovic et al., 2019; Middleton and Ortiz-Catalan, 2020).

As direct haptic feedback in neural prosthesis further develops, alternative methods to reintroducing haptic feedback should be considered. An important question with regards to establishing an effective non-direct form of haptic feedback for amputees is how to stimulate a sense of touch on a limb that has been removed. Given that prosthesis users do not have the possibility for local feedback (if not innervated) the purpose of this research was to determine if proximal feedback, i.e., feedback administered to a upper arm residual, would also allow for the induction of sense of embodiment. Therefore, we investigated how converting natural haptic feedback from the fingertips into vibrotactile feedback and administering this vibrotactile feedback to another location on the body may allow participants to experience haptic feedback without its natural delivery to their fingertips. To measure how these manipulations affect participants, we used a virtual hand illusion task to first assess if participants' sense of embodiment of a virtual hand changes after controlling a virtual hand that has been spatially shifted. Then, we investigate how converting natural haptic feedback from the prosthetic fingertips into vibrotactile feedback administered to another location on the body may allow participants to experience haptic feedback and if and how this experience affects embodiment. If sense of embodiment is not negatively impacted when switching from natural feedback to proximal vibrotactile feedback, proximal vibrotactile feedback should be further studied and considered when designing prostheses.

### Vibrotactile and Proximal Feedback

Vibrotactile feedback is an excellent option for reenabling the haptic feedback of prosthetics (Pylatiuk et al., 2006; Chatterjee et al., 2008; Stepp and Matsuoka, 2012). Using vibration feedback has been demonstrated to have improvements over using vision alone as a feedback (Clemente et al., 2016; Rosenbaum-Chou et al., 2016; Yamada et al., 2016). Raveh et al. (2018) created a task where participants had a myoelectric-controlled hand attached to their right arm. The attached hand had pressure sensors that triggered vibrotactile feedback in motors attached to the participant's upper arm. When vibrotactile feedback was enabled and visual acuity was limited in a dark room during a Box and Blocks task, participants completed the task more quickly and with fewer errors than when the vibrotactile feedback was not enabled. In addition to functional improvement through vibrotactile feedback, D'Alonzo et al. (2015) found strong evidence that vibrotactile feedback promotes embodiment using the rubber hand illusion with amputee participants who had phantom sensations. The authors recruited participants who had phantom sensations of fingers that had drifted onto mechanoreceptors on their residual limb and mapped and applied vibrotactile stimulators to the finger-mapped areas. They found significant differences in questionnaire results, proprioceptive drifts, as well as skin conductance responses when vibrotactile feedback was given synchronously to the appropriate phantom sensation areas vs. asynchronous feedback to those areas. This is a promising example of vibrotactile stimulation facilitating strong embodiment of an alien limb, but it remains to be seen how strong sense of embodiment can be promoted when tactile receptive fields have not already migrated to specific areas on the residual limb.

Few rubber hand illusion related studies have investigated how manipulating the location of haptic feedback can affect the illusion. Riemer et al. (2014) found that stroking fingers on the obscured hand spatially incongruent with the fingers on the rubber hand eliminated the effects of the illusion. This suggests that changing the location of haptic feedback in the rubber hand illusion may result in decreased embodiment. However, there is evidence that stroking incongruent receptive fields on the back of the obscured hand during the rubber hand illusion does not significantly reduce embodiment unless it is also coupled with postural mismatch (Costantini and Haggard, 2007), which is an important example of changing location of haptic feedback without reducing embodiment.

### Objectives and Hypotheses

To assess how different haptic feedback locations (local vs. proximal) and modalities (natural vs. vibrotactile) affect proprioceptive drift, we created a virtual rubber hand illusion task in which participants grasp an object in VR. Detailed hand tracking and virtual collision detection were used to activate small vibrotactile motors attached to participant's fingertips and upper arm to provide haptic feedback for the virtual hand at different body locations while keeping visual feedback for grasping constant.

The goal of this study was to replicate a rubber hand illusion effect in VR to explore how changing the site and modality of haptic feedback affects sense of embodiment. We used a virtual hand illusion task in VR in conjunction with five different haptic feedback conditions (Natural, Natural + Local Vibratory, Local Vibratory, Proximal Vibratory, and No Haptic Feedback) to evaluate the overall sense of embodiment associated with a virtual limb. Keeping visual feedback constant, we modulated both the type and location of haptic feedback. We hypothesized that measurements reflecting the strength of the sense of embodiment of the virtual arm would increase significantly, relative to the sense of embodiment of the virtual arm before each condition. We also expected measurements reflecting the strength of the sense of embodiment of the virtual arm to differ across types of feedback. More specifically, we expect feedback conditions involving the natural feedback from touch (Natural, Natural + Local Vibratory) to induce a stronger illusion than the three conditions that did not. Finally, we also expected measurements reflecting the strength of the sense of embodiment of the virtual arm to be the same or less when changing the feedback location from local to proximal.

## Materials and Methods

### Participants

Participants were recruited through the UC Psychology Research Participation System (SONA Systems, Tallinn, Estonia). Participants were screened for right handedness and were asked to wear contacts rather than glasses to avoid discomfort from the head mounted display. In total, 30 participants were recruited of which 5 participants were excluded due to hardware or software malfunction. A total of 25 participants [14 females; mean age = 19.12 years, standard deviation (*SD*) = 1.30] were ultimately included in data analysis. To oblige the University of Cincinnati COVID-19 restrictions, researchers and participants wore masks at all times during the experiment; all surfaces and non-disposable equipment were regularly cleaned and disinfected before and after each participant. Headsets have been disinfected and put on a 48 h rotation before reuse. Furthermore, researchers always kept a 10ft minimal distance from participants, which required participants to don the experimental equipment by themselves following verbal instructions by the experimenter. This study is aligned with and covered by the University of Cincinnati Institutional Review Board Protocol #2012-2827. All participants read and signed an informed consent form before engaging in the experiment.

### Apparatus

**Figure 1:**
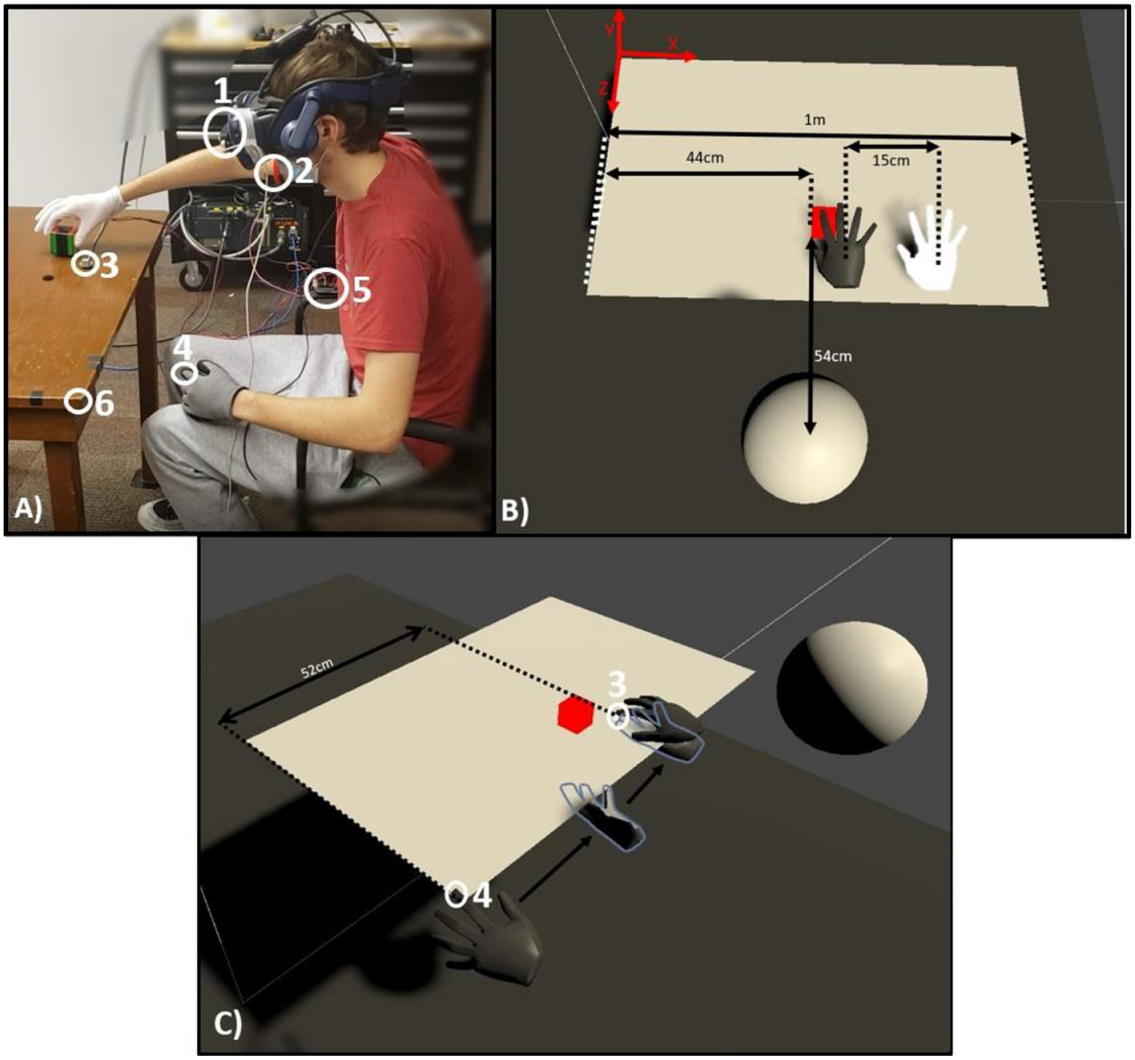
Experimental setup. **(A)** A participant during the natural feedback condition. (A1) Leap Motion sensor; (A2) vibrostimulator armband; (A3) fixed Polhemus sensor; (A4) reference Polhemus sensor attached to tip of index finger; (A5) Arduino controlling vibration signals; (A6) table corner used to reset left finger at the start of each proprioceptive estimate. **(B)** A top-down depiction of what participants viewed in VR. The white hand represents the actual location of the participant's right hand while the dark hand is its visible position shifted 15 cm. **(C)** Over the shoulder depiction of a proprioceptive estimate. (C3) Fixed Polhemus sensor; (C4) reference Polhemus sensor attached to tip of index finger. Arrows show the reference sensor as it travels from the start of the proprioceptive estimate, to midway through the estimate, and at its final resting point when the participant declares they are satisfied with their estimate. During the estimate, the participant's headset is blacked out, so they are not viewing either hand in the virtual space.

The experiment took place in an aligned virtual and physical environment with a participant in first person view sitting at a table (see Figure 1). A 5 cm^3^ cube was available in the physical space and its dimensions were the same as the cube available on the virtual table. The VR environment was created in Unity 2020.1.7 (Unity Studios, San Francisco CA) and enacted with an HTC Vive Pro headset (HTC Corporation, Bellevue WA). A Leap Motion (San Francisco CA) hand tracking system was integrated with the VR environment. The Leap Motion tracking sensor was attached to the front of the headset. Two wired Polhemus Patriot (sampling rate 60 Hz, Alken Industries, Ronkonkoma NY) sensors were attached to the table and the participant's left index finger for proprioceptive drift assessment.

Vibrotactile stimulation has previously been used to successfully induce the virtual hand illusion (Kokkinara and Slater, 2014; Padilla-Castañeda et al., 2014). We therefore created a glove with one vibrotactile motor (Tatoko 10 mm × 3 mm Mini Vibration Motor DC 3V 12,000 rpm Flat Coin Button-Type) on the tip of the right index finger, and one on the tip of the right thumb. In addition, participants were asked to don an armband around their upper arm with one vibrotactile motor on their biceps and one on their triceps. The motors were driven by an Arduino Uno which were activated when the appropriate finger collided with the virtual cube in Unity.

### Procedure

Participants were seated at the experiment table, where they read and signed a consent form. They were then given instructions on how to place the vibrotactile motors. First, they pulled the disposable armband over the center of the upper right arm. They adhered one vibrotactile motor to the center of the biceps, and a second motor 180 around the arm on the triceps. They then donned a pair of disposable gloves and adhered one vibrotactile motor to the tip of their right index finger, and a final motor to the top of their right thumb. Finally, they wore a reusable glove on top of the disposable glove on their left hand, which had one of the two Polhemus sensors (*reference sensor*) attached to its index fingertip.

After all upper limb equipment was correctly attached, participants were directed to the Polhemus sensor fixed to the table in front of them (*fixed sensor*). They were told that they had to repeatedly grasp the cube that was placed on the table in front of them and that during before and after each grasping task, they would need to place their right index finger on top of the sensor while their vision was obscured. Next, participants were asked to put on the Vive Pro headset.

Once the headset was turned on, participants found themselves in a VR space with a virtual table positioned in the exact location relative to the physical table in front of them (see Figure 1). The real cube was also secured on the right side of the table and was represented by a virtual cube in the same location in VR. The participant's right hand was tracked using the Leap motion sensor and made visible as a virtual hand. This pre-testing VR space was used to explain the details of the experiment before initiating data collection.

During each of the 5 conditions, the task was to grasp and release the virtual cube. A metronome clicking at 1 Hz per second was played through the Vive Pro earphones, and participants were instructed to repeatedly open and close their right hand in order to grasp the cube at a rate matching the metronome clicks for 2 min, resulting in 120 grasping motions per condition. Importantly, after the initial pre-testing space (where the virtual right hand was positioned in the same location as the real right hand), the virtual hand's position was shifted 15 cm to the left during all grasping phases (see Figure 1B). The 15 cm-shift is a distance that is considered within the optimal window to induce the rubber-hand illusion (Lloyd, 2007; Davies et al., 2013; Kalckert and Ehrsson, 2014).

The experiment consisted of 5 conditions in which the type of haptic feedback (natural, natural + local vibrotactile, local vibrotactile, proximal vibrotactile, and no tactile feedback) was manipulated. In all conditions besides “proximal vibrotactile” and “no tactile feedback,” haptic feedback was administered locally to the fingertips. In the natural haptic feedback condition, a real cube was placed in the position of the virtual cube under the right hand to be grasped. In the natural + vibrotactile condition, the real cube was placed in the position of the virtual cube and the participant received haptic feedback through the vibration motors in the glove whenever the fingers reached and passed through the virtual cube. In the local vibrotactile condition, the participant received haptic feedback through the vibration motors in the glove whenever the fingers reached and passed through the virtual cube. The local vibrotactile feedback condition alone allowed us to investigate if local vibrotactile feedback was sufficient to drive the illusion. The natural feedback + local vibratory allowed us to further investigate if there were additional benefits or detriments from local vibratory feedback relative to natural feedback. In the proximal vibrotactile condition, the participant received haptic feedback through the vibration motors in the armband whenever the fingers reached and passed through the virtual cube. Finally, in the no haptic feedback condition, participants repeatedly grasped the virtual cube with no haptic feedback provided. Conditions were block-randomized within participants to decouple the effect of feedback type on embodiment from effects of repeated exposures across conditions.

At the start of each condition, participants were prompted to give an initial estimation of the perceived location of their right index finger (proprioceptive estimate). To this end, the Vive screen was blacked out, and participants were instructed to bring their left index finger with the reference sensor attached to the reset position, which was the left corner of the table (see Figure 1C). Then, they were instructed to bring their hand under the table and in one fluid motion place the reference sensor against the bottom of the table where they believed the tip of their right index finger, which was placed upon the fixed sensor. They verbally declared when they were confident with their estimate, and the researcher stopped and saved the recording of the two sensors positions. This proprioceptive estimate assessment was performed three times before each condition, and 3 times after each condition, resulting in three pre-grasping and three post-grasping estimate recordings.

After each condition's post-grasping proprioceptive estimates, participants were instructed to carefully remove the headset, and answer the virtual hand embodiment questionnaire. The questionnaire contained nine questions adapted from Longo et al. (2008) and Huynh et al. (2019) and was designed to assess the strength of the subjective experience of virtual hand embodiment (see Table 1). Answers to each question were collected using a seven-point scale ranging from strongly disagree to strongly agree. The questionnaires were given to participants on paper to help reset the effects of the illusion in between conditions, as participants re-adapted toward their natural felt location of their right hand by holding and using a pen.

**Table 1:** Questionnaire descriptive statistics.

	**While grasping the cube…**	**Category**	**Mean of 7-point Likert scale**	**Standard error**
1	…it seemed like I was looking directly at my own hand, rather than a virtual hand	Ownership	5.24	0.23
2	…it seemed like the virtual hand began to resemble my real hand	Ownership	5.87	0.20
3	…it seemed like the virtual hand belonged to me	Ownership	5.89	0.20
4	…it seemed like the virtual hand was my hand	Ownership	5.49	0.22
5	…it seemed like the virtual hand was part of my body	Ownership	5.47	0.18
6	…it seemed like my hand was in the position where the virtual hand was	Location	6.04	0.23
7	…it seemed like the virtual hand was in the position where my hand was	Location	6.20	0.19
8	…it seemed like I could have moved the virtual hand if I had wanted	Agency	6.27	0.20
9	…it seemed like I was in control of the virtual hand	Agency	6.40	0.18

*Items adapted from Longo et al. (2008) and Huynh et al. (2019)*.

## Data Analysis and Results

### Data Preparation

Each participant was recorded with three pre-grasping and three post-grasping estimates before and after each of the five feedback conditions, yielding 30 total recordings. The final 10 samples of both position time series in x-direction (see Figure 1B for a depiction of XYZ coordinate planes) were isolated and averaged in each recording, resulting in one Offset value per trial. The X coordinate of the reference sensor was subtracted from the X coordinate of the fixed sensor, resulting in the *Offset* value for each estimate assessment.

The Offset reflects the difference value on the X axis between the participant's right index finger and where they have placed their left index finger under the table (see Figure 1C). An Offset of 0 means they were able to perfectly line up their two index fingers with the table in between. A negative Offset means they placed their left index in the direction of the virtually shifted hand with respect to the fixed sensor, and a positive Offset means they placed their left index finger to the right of the fixed sensor (see Figure 1A). We define the *Proprioceptive Drift* as the difference between the pre-grasping offsets and the post-grasping Offsets.

The manipulation that was constant across all conditions was the shift imposed on the virtual location of the hand; the virtual hand was visually shifted 15 cm to the left with respect to the real hand (in the more traditional rubber hand illusion our real hand would be referred to as the “obscured” hand). As such, participants received visual feedback of their hand that was shifted 15 cm to their left, which in line with the rubber-hand illusion theory, should drive the illusion and by extension their proprioceptive reports toward the left. In other words, we expected post-grasping Offsets to be more negative (shifted more toward the virtual hand) than pre-grasping Offsets.

We therefore analyzed the Offset values for each participant to determine if Offset systematically varied as a function of the *Estimate Block* (Pre-grasping, Post-grasping), *Feedback Type* (Natural, Natural + Vibratory, Vibratory, No Haptic Feedback), and *Feedback Location* (Local, Proximal, No Haptic Feedback).

We performed a sensitivity analysis following the procedures described by Westfall et al. (2014), using the web-based app (Pangea) developed by the first author and available on his webpage (https://jakewestfall.shinyapps.io/pangea/). Results showed that considering our sample size (*n* = 25) and the number of estimate attempts per condition (3), the mixed-effect analysis we employed had 80% power to detect moderate effect sizes: Cohen's *d* > 0.30 for test condition effect and Cohen's *d* > 0.35 for test condition × feedback type and test condition × feedback location.

### Feedback Type

To determine if feedback type affects embodiment, we submitted the Offset values to a linear mixed model (LMM) analysis (R package: lmerTest) with *Estimate Block and Feedback Type* as fixed effects. Analyses of the descriptive statistics suggested an effect for the repeated measures taken on the Offset values; thus, we included *Estimate Attempt* (First, Second, Third) in the model as a control variable. To control for inter-individual variability, *Participant* was entered into the model as random effect. We excluded outlier data with a standardized residual distance <2.5 standard deviations from the average standardized error. Seven data points which comprised ~1.2% of total number of data points were excluded.

The results revealed a significant main effect of Estimate Attempt on the Offset values, *F*_(2,540.95)_ = 25.05 *p* < 0.0001. Pair-wise comparisons with Tukey correction showed that Offsets were significantly greater (more shifted away from the virtual hand) for Second (*M* = 1.14 cm, *SE* = 0.28) and Third (*M* = 1.47 cm, *SE* = 0.27) estimates compared to First estimates (*M* = 0.34 cm, *SE* = 0.26), all *p*s <0.001; there was no significant difference between Second and Third estimates, *p* = 0.13.

**Figure 2:**
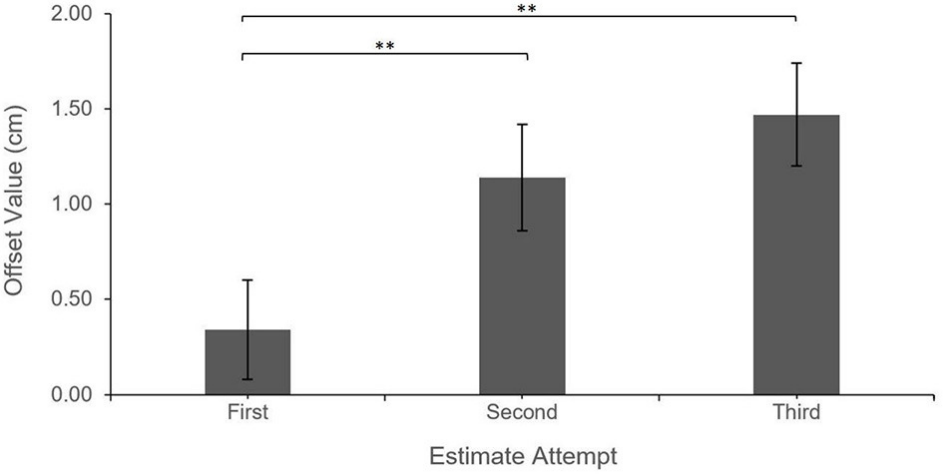
Feedback type Offset by estimate attempt. Error bars reflect the standard error. ***P < 0.001; **P < 0.01.

**Figure 3:**
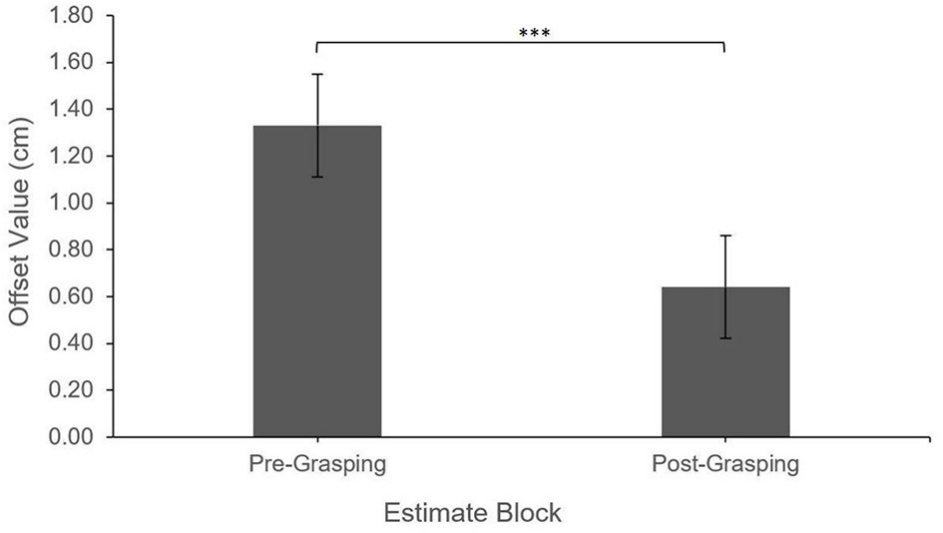
Feedback type Offset by estimate block. Error bars reflect standard error. ***P < 0.001.

Additionally, after controlling for Estimate Attempt, there was a significant main effect of Estimate Block (Pre-grasping vs. Post-grasping), *F*_(1,540.96)_ = 26.06, *p* < 0.0001. Offset values were greater (more shifted away from the virtual hand) for the Pre-grasping estimates (*M* = 1.33 cm, *SE* = 0.22) when compared to those made Post-grasping (*M* = 0.64 cm, *SE* = 0.22), indicating a shift in the perceived position of the real hand toward the virtual hand; this effect occurred regardless of Feedback Type, i.e., all interaction effects were non-significant, all *p* > 0.33. See Figures 2, 3 for illustration of described effects.

### Feedback Location

To determine if the location of feedback moderates the effect of embodiment, we submitted the Offset values to a second linear mixed model (LMM) analysis (R package: lmerTest) with *Estimate Block* and *Feedback Location* as fixed effects. Again, analyses of the descriptive statistics suggested an effect for the repeated measures on the Offset values; thus, we included *Estimate Attempt* (First, Second, Third) in the model as a control variable. To control for inter-individual variability, *Participant* was entered into the model as random effect. We excluded outlier data with a standardized residual distance <2.5 standard deviations from the average standardized error. Twelve data points which comprised ~2.9% of total number of data points were excluded.

The results revealed a significant main effect of Estimate Attempt on the Offset values, *F*_(2,390.80)_ = 22.76, *p* < 0.0001. Pair-wise comparisons with Tukey correction showed that Offsets were significantly greater (more shifted away from the virtual hand) for Second (*M* = 1.10 cm, *SE* = 0.32) and Third (*M* = 1.42 cm, *SE* = 0.30) estimates compared to First estimates (*M* = 0.21 cm, *SE* = 0.30), all *p*s <0.001; there was no significant difference between Second and Third estimates, *p* = 0.21.

**Figure 4:**
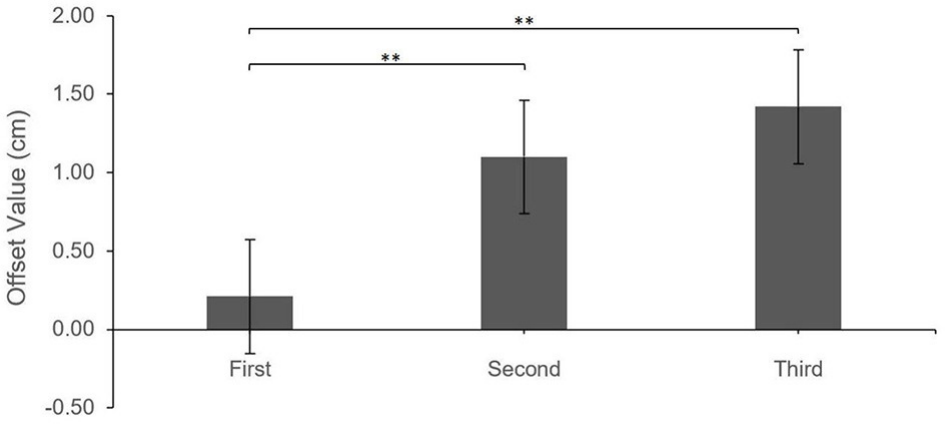
Feedback location Offset by estimate attempt. Error bars reflect standard error. ***P < 0.001; **P < 0.01.

**Figure 5:**
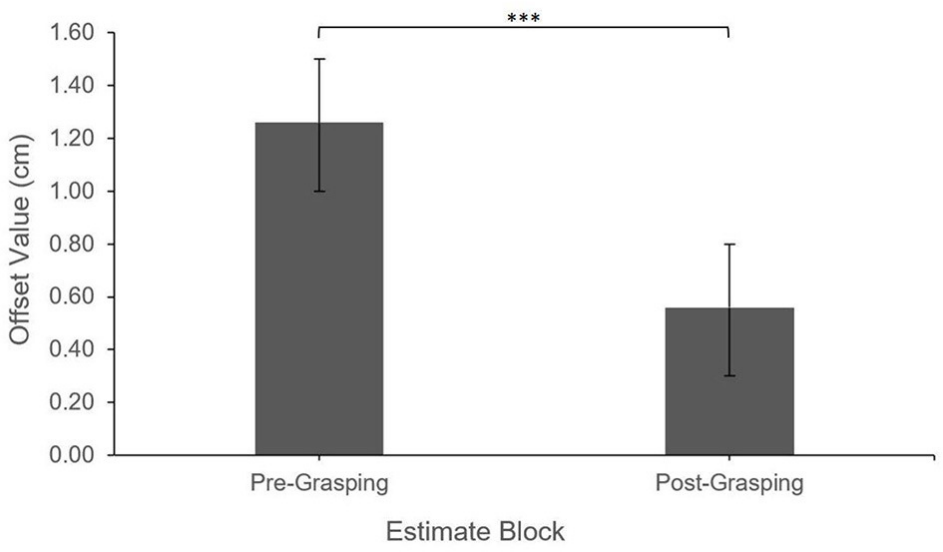
Feedback location Offset by estimate block. Error bars reflect standard error. ***P < 0.001.

Additionally, after controlling for Estimate Attempt, there was a significant main effect of Estimate Block (Pre-grasping vs. Post-grasping), *F*_(1,390.91)_ = 19.21, *p* < 0.0001. Offset values were greater (more shifted away from the virtual hand) for the Pre-grasping estimates (*M* = 1.26 cm, *SE* = 0.24) when compared to those made Post-grasping (*M* = 0.56 cm, *SE* = 0.26), indicating a shift in the perceived position of the real hand toward the virtual hand; this effect occurred regardless of Feedback Location, i.e., all interaction effects were non-significant, all *p* > 0.16. See Figures 4, 5 for illustration of described effects.

### Questionnaire Scores

**Figure 6:**
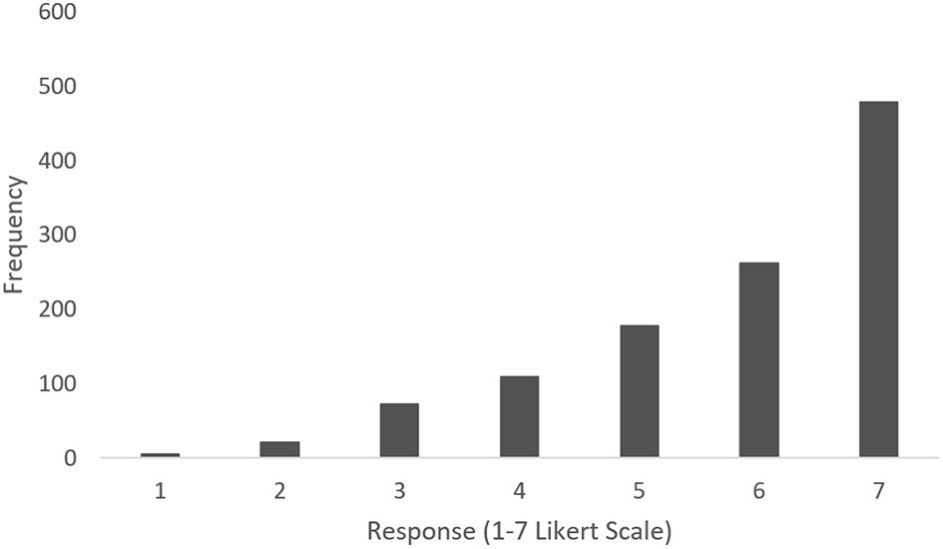
Overall questionnaire response frequencies across all questions and conditions.

Questionnaire scores were extremely skewed toward high scores (see Figure 6). A chi-square test of independence was performed to examine the relation between condition and question response. Question responses were formed by condensing Likert scores into Disagree (scores 1–3) Neutral (score 4), and Agree (scores 5–7). The relation between condition and question response was not significant, $X_{\left(8,\text{N}=1,125\right)}^{2}$ = 14.49, *p* < 0.05.

Questionnaire scores were broken down into their established categories of Ownership, Perceived Location, and Agency (see Table 1). Pearson's product-moment correlations were run to compare questionnaire scores to Proprioceptive Drifts across the five feedback conditions. Questionnaire responses for Ownership did not correlate with Proprioceptive Drifts across conditions, *r*_(123)_ = −0.26, *p* = 0.79. Questionnaire responses for Perceived Location did not correlate with Proprioceptive Drifts across conditions, *r*_(123)_ = 0.02, *p* = 0.84. Questionnaire responses for Agency did not correlate with Proprioceptive Drifts across conditions, *r*_(123)_ = −0.10, *p* = 0.92.

## Discussion

While we plan to work with amputee populations with robotic hands in the future, VR serves as a useful tool to investigate the utility of various forms of feedback as well as the sense of embodiment. An eventual goal in prosthetics is to restore natural feelings to both control and feedback of the prosthesis. Working with non-amputees in VR allows use to directly compare natural forms of haptic feedback to potential alternatives. In the current experiment, we assessed if participants' sense of embodiment of a virtual hand changes after controlling a virtual hand that has been spatially shifted. We then investigated the impact of different feedback types and feedback locations on embodiment of a virtual hand. Participants repeatedly grasped a cube in VR, while the actual haptic feedback they received was manipulated. They either grasped only a virtual cube, grasped the cube in VR while simultaneously grasping the real cube, or received vibratory feedback on their fingertips or on their upper arm.

Analyzing Proprioceptive Drift derived from Estimate Offsets for both haptic Feedback Type and Feedback Location, we found a significant main effect for Estimate Block, indicating a significant proprioceptive drift toward the shifted virtual hand when comparing pre-grasping Offsets to post-grasping Offsets. However, the drift was equally present across all feedback conditions and feedback locations, with no difference between them. It is important to note that the present study is powered to detect moderate-sized effects. Thus, it is possible that we have missed small effects of feedback type and location that might exist. Results are conclusive in suggesting, however, that embodiment effects were observed regardless of the experimental condition. In general, the existence of a significant drift shows that the virtual hand illusion was successfully replicated in our VR environment. Furthermore, our results suggest that participants' proprioceptive feedback (through the grasping activity) coupled with the visual feedback was enough to facilitate the illusion and was not further affected by additional forms of feedback. This is in line with a recent similar study by Huynh et al. (2019) who found that synchronous motor feedback is sufficient to induce a rubber-hand illusion.

Besides this, Huynh et al. (2019) also showed that sense of embodiment is strengthened as more types of feedback are combined, even demonstrating that synchronous feedback in one modality appeared to compensate for asynchronous feedback in the other. We were surprised to find that combining feedback types in our study did not enhance the sense of embodiment of the virtual hand over the no haptic feedback condition, which resulted in a similar drift as the other four conditions. This seems to suggest that the visual feedback coupled with motor feedback from control of the virtual hand were sufficient to drive the effect of the illusion. As our aim for his study was not to explore the impact of motor feedback, but the impact of dislocated feedback, we will have to leave the exploration of this discrepancy (by for example including a no-motor condition) to future research.

Besides the presence of the proprioceptive drift effect, we also found a significant main effect for Estimate Attempt for both feedback type and location, indicating that proprioceptive estimates trended to the right (the opposite direction of the visual shift of the hand) as participants iterated through each set of three estimates. This may reflect the elasticity of the illusion, with the estimate immediately following the grasping phase resulting in the highest drift toward the virtual hand and a quick temporal decline of the effect afterwards.

In addition to the quantitative assessment of proprioceptive drift as an indicator of sense of embodiment, we also used a modified embodiment questionnaire to assess the subjective experience of participants. Answers in all categories were extremely skewed toward high scores, with all questions averaging at least five (Somewhat Agree) on the seven-point Likert scale. Questionnaire scores did not significantly differ across feedback condition. Like the proprioceptive estimate findings, these questionnaire response findings suggest that participants' proprioceptive feedback (through the grasping activity) coupled with the visual feedback was enough to facilitate the illusion and was not further affected by additional forms of feedback. The fact that the scores were extremely skewed toward the affirmation of embodiment might be due to the fact that in immersive environments, such as VR, the only available visual feedback is entirely provided by the simulation. Hence, we might have a natural tendency to trust the provided visual feedback, especially with respect to relative position without additional frames of reference. While our primary interest is improving the sense of embodiment of a virtual hand in order to extend these findings to improve prosthetics, we are also interested in how sense of embodiment of a virtual body can impact the quality of VR products and training programs. Vibrotactile feedback is already used within controllers of modern VR systems to signal different types of interactions. Further research on how types and locations of haptic feedback affect the embodiment and immersion of a virtual body will enable the enhancement of a myriad of VR tools and products.

When questionnaire scores were broken down into their established categories of Ownership, Perceived Location, and Agency and correlations were run between each category's questionnaire scores and proprioceptive drift values across feedback conditions, none of the three categories' scores correlated with proprioceptive drifts. The evident lack of correlation between questionnaire scores and proprioceptive drift measurements, which was already documented by Rohde et al. (2011), renders the fact that most rubber hand illusion research considers questionnaire responses and proprioceptive drift to be equally as valid assessments of embodiment, surprising. Together with the fact that we were able to show that even the illusion as expressed in drift assessment vanishes quickly, we overall may need to consider alternative paradigms if we want to gain a true assessment of prosthetic embodiment.

In this context, there may be limitations in applying our findings from VR to the improvement of real-world prosthetics. D'Alonzo et al. (2019) found that vibrotactile feedback can reliably induce a virtual hand illusion. However, they also found evidence suggesting that vibrotactile feedback applied in a VR setting induces a stronger illusion as compared to a vibrotactile feedback in a real rubber hand setting. This finding indicates that when moving from virtual environments to world applications real, vibrotactile feedback is less effective in impacting embodiment.

There are also limitations relating to several optimizations of the virtual hand illusion that were not implemented in our setup that may have enhanced the strength of the illusion and highlighted more differences between feedback conditions. Body continuity is a significant factor in the strength of the rubber hand illusion that was not implemented in our setup but may have increased the strength of the illusion (Perez-Marcos et al., 2012; Tieri et al., 2015). The level of realism and congruence in appearance can also impact the strength of the illusion, with gender (Schwind et al., 2017), race (Lira et al., 2017), and overall appearance (Pyasik et al., 2020) each impacting measurements of sense of embodiment when manipulated. We would have likely increased the strength of the illusion along with its impact on sense of embodiment if we had added a wrist and arm to the virtual hand, along with improving the realistic appearance of the hand and adjusting its gender and race for each participant. Nevertheless, we believe that despite the lack of differences found among the conditions in this study, we are encouraged by the proprioceptive drift driven by the experimental task. Furthermore, it is encouraging that converting local feedback to proximal does not reduce the effect of the illusion. Another option for future studies would be to incorporate an asynchronous feedback condition to determine if the proprioceptive drift observed in the present synchronous feedback conditions persists (or even increases) as has been observed in other studies (D'Alonzo et al., 2019).

## Conclusion

In this study we investigated the feasibility of proximal vibrotactile feedback for inducing embodiment of a virtual hand. We showed that neither the location of feedback induction, nor the changed modality limited the induction of proprioceptive drift as a measure of induced embodiment of the virtual hand. These findings might have a large impact on the future of prosthetics and all wearable devices as adding proximal, vibrotactile feedback is less invasive and risky than neural connection while still providing closed loop feedback in grasping activities and subsequent sense of embodiment, which may in turn enhance prosthesis acceptance rates. Finally, proximal vibrotactile feedback is a low-cost solution and may be added to existing prosthetic systems by simply retrofitting (or creating new ones) using relatively simple technology.

## Data Availability Statement

The raw data supporting the conclusions of this article will be made available by the authors, without undue reservation.

## Ethics Statement

The studies involving human participants were reviewed and approved by University of Cincinnati Institutional Review Board/Human Research Protection Program. The patients/participants provided their written informed consent to participate in this study.

## Author Contributions

CM, TL, KS, and PS contributed to conception and design of the study. CM collected data and wrote the first draft of the manuscript. RM contributed to experiment setup creation. CM, SC, and PS contributed to statistical analyses. SC and TL wrote sections of the manuscript. All authors contributed to manuscript revision, read, and approved the submitted version.

## Conflict of Interest

The authors declare that the research was conducted in the absence of any commercial or financial relationships that could be construed as a potential conflict of interest.
